# The Status of *Posidonia oceanica* at Tremiti Islands Marine Protected Area (Adriatic Sea)

**DOI:** 10.3390/biology11060923

**Published:** 2022-06-16

**Authors:** Andrea Tursi, Francesco Mastrototaro, Federica Montesanto, Francesco De Giosa, Anna Lisco, Antonella Bottalico, Giovanni Chimienti

**Affiliations:** 1 Department of Biology, University of Bari Aldo Moro, 70125 Bari, Italy; francesco.mastrototaro@uniba.it (F.M.); annalisco87@gmail.com (A.L.); antonella.bottalico@uniba.it (A.B.); giovanni.chimienti@uniba.it (G.C.); 2 CoNISMa (Consorzio Nazionale Interuniversitario per le Scienze del Mare), 00196 Rome, Italy; 3 Institute of Agriculture and Natural Resources, University of Nebraska-Lincoln, 1400 R Street, Lincoln, NE 68588, USA; federica.montesanto@uniba.it; 4 Environmental Surveys S.r.l. (ENSU), Via De Gasperi, 74123 Taranto, Italy; francescodegiosa@ensu.it

**Keywords:** seagrass, meadows protection, marine phanerogam conservation, seagrass monitoring, sensitive habitats, coastal management, marine protected area management, scientific diving, infralittoral habitat, Mediterranean Sea meadows

## Abstract

**Simple Summary:**

The seagrass *Posidonia oceanica* is the most important marine phanerogam of the Mediterranean Sea due to its meadows’ complexity, persistence, and extension. These habitats provide a suite of ecosystem goods and services, being of primary importance in marine conservation. Despite their central role in the coastal ecology, *P. oceanica* meadows are undergoing overall deterioration and fragmentation in the basin mostly due to anthropogenic impacts at local to global scales. In the last decades, several management measures have been proposed aiming to improve the meadow health conditions, while the periodic monitoring of *P. oceanica* meadows allows for verifying their effectiveness. Here, we report the results of the monitoring of *P. oceanica* at Tremiti Islands Marine Protected Area (Adriatic Sea, Italy) carried out in 2003, 2015, and 2020. A general worsening was observed, particularly enhanced by direct anthropogenic impacts mostly related to anchoring practices, as well as by a certain level of sedimentation possibly deriving from coastal development. However, the identification of these impacts and the correct management of human activities to mitigate them produced positive results in a relatively short time span.

**Abstract:**

*Posidonia oceanica* meadows are Mediterranean coastal habitats of great conservation importance. This study is focused on a meadow located at Tremiti Islands Marine Protected Area (Adriatic Sea, Italy), which was monitored in 2003, 2015, and 2020 to evaluate its health state over time in relation to coastal human activities, which have been highly affecting this MPA for the last 20 years. To assess any change in the physiognomy of the meadow, rhizome density, percentage coverage, and lower limit progressions and/or regression over time were evaluated by scuba diving, while the distribution and extension of the meadow were assessed through habitat mapping using a side-scan sonar. Moreover, phenological and lepidochronological analyses were performed on the collected rhizomes to assess the leaf area index (LAI, m^2^m^−2^) and the rhizome age (lepidochronological years). Our study showed a general deterioration of *P. oceanica* meadow from 2003 to 2020, with a significant reduction of its absolute and relative rhizome density and LAI at almost all sampling stations, absence of renovation of the meadow, and lower limit regression and overall worsening of the main conservation status indicators. However, appropriate management actions, such as the establishment of mooring buoy fields, supported the improvement of the *P. oceanica* status at the local scale with a significant increase in density and LAI and the presence of active stolonization processes, suggesting that mitigation actions can play a crucial role in the conservation of this habitat. On the contrary, local anthropogenic impacts, especially anchoring and coastal development, markedly affect the resilience of *P. oceanica* meadows to global stressors, such as climate change.

## 1. Introduction

*Posidonia oceanica* (L.) Delile, 1813, is a Mediterranean-endemic phanerogam, widespread all over the basin and able to form extensive meadows, thus considered an ecosystem engineer of the coastal zone [1,2]. *P. oceanica* meadows represent one of the richest habitats in the infralittoral Mediterranean seabed, mostly common between 5 and 30 m depth, although they can be present from 1 to 50 m depth depending on seawater characteristics (e.g., transparency of the water, nutrient concentration, sedimentation rate, temperature) [3,4]. *P. oceanica* can live on both sandy and rocky bottoms, where it forms meadows of high ecological value [4].

Healthy meadows provide many ecosystem goods and services, representing habitats of great conservation importance. They are valuable hotspots of biodiversity, hosting about 25% of the Mediterranean marine species, which rely on these habitats as spawning, nursery, feeding, and refuge areas [3,5,6,7]. Moreover, due to the high photosynthetic rates, *P. oceanica* absorbs large amounts of CO_2_ (up to 426.6 g C m^−2^) [8] and releases a high quantity of O_2_ (up to 20 L of O_2_ per m^2^) [9], antagonizing the ocean acidification and the greenhouse effect. Finally, *P. oceanica* meadows successfully stabilize the seabed and contrast coastal erosion through the edification of biogenic three-dimensional structures formed by old leaves, roots, and rhizomes (*mattes*), as well as through the formation of leaf heaps beached along the seashore (*banquettes*) [10,11,12,13,14,15,16].

In the last decades, this sensitive habitat has been suffering an alarming deterioration all over the Mediterranean Sea, especially in its western area, with a strong areal contraction and a marked reduction of bundle density, together with a bathymetric rise in the lower limit, from about 30 to 15–20 m [17,18,19,20,21]. This reduction is mainly caused by human activities, determining direct and indirect mechanical impacts on the meadows, such as coastal development, bottom-contact fishing gears [7,22], and anchoring [14,23,24,25]. Moreover, intensive aquaculture activities and coastal dumping of pollutants can determine harmful eutrophication events [26,27]. Other human-related causes of the decline of *P. oceanica* meadows include the spread of competitive alien species [23,28], mucilaginous blooms, and global warming [29,30]. Furthermore, *P. oceanica* relies almost exclusively on stolonization (vegetative reproduction), while sexual reproduction is less frequent than 10% of the total reproductive events [30,31,32,33,34]. This biological feature reflects very low genetic variability, leading to a limited recovery capacity after natural and/or anthropic disturbances [35]. However, according to some recent studies, the meadow deterioration has been slowing down in the last years, likely because of management interventions, confirming the strong influence of coastal human activities on *P. oceanica* meadows’ health state [36,37,38].

The awareness of the strong degradation of these habitats has led, in the last three decades, to the proposal of several protection and management measures targeting *P. oceanica* meadows, which now represent one of the most regulated and monitored habitats in the Mediterranean Sea [2,39]. The EU Habitat Directive (92/43/EEC) lists this species as a priority for conservation, whose monitoring is mandatory. *P. oceanica* has also been considered a reliable bioindicator by the EU Marine Strategy Framework Directive (2008/56/EC) due to its sensitivity to anthropogenic impacts and environmental changes [37,40,41,42,43,44,45]. Based on the main characteristics of *P. oceanica*, several ecological indexes have been developed to assess the meadow conservation status and, consequently, the environmental status [38,42,43]. In fact, parameters such as meadow density (number of rhizomes per square meters), amount of leaf surface of the meadow (leaf area index (LAI)), rhizome growth rate and age, and lower limit dynamics represent valuable indicators of the health state of *P. oceanica* meadows, thus being widely used in ecological indexes. 

Relevant examples of the most used indexes in the Mediterranean basin are the classifications proposed by Giraud [46] and by Pergent and colleagues [47] in the second half of the last century, as well as, more recently, the POSID [48], POMI [49], Valencian CS [50], PREI [38], and BiPo [51], used to assess the health state of the meadows [52] and to quantify the ecological quality ratio (EQR) to evaluate the status of a monitored site in comparison with pristine conditions [38,44,47,53]. 

Due to its importance, many marine protected zones, such as marine protected areas (MPAs) and marine sites of community importance (SCIs), have been historically identified based on the presence of *P. oceanica* meadows throughout the basin. This is the case of the Tremiti Islands MPA (Adriatic Sea), instituted more than 30 years ago to preserve, among the others, the northernmost Adriatic meadow of *P. oceanica* along the Italian coast together with a suite of vulnerable marine ecosystems [54,55,56].

This study is focused on the monitoring of *P. oceanica* meadow at Tremiti Islands MPA carried out in 2003, 2015, and 2020. Although genetic data are still lacking, this meadow is considered almost monoclonal due to the scarce to null occurrence of sexual reproduction events to the best of our knowledge, thus being particularly sensitive to anthropogenic impacts. This meadow is considered a fundamental habitat of the infralittoral zone of the Tremiti Archipelago, enhancing the biodiversity and the richness of this MPA [57]. Both in situ and ex situ studies, including habitat mapping, rhizome density assessment, lower limit monitoring (*balisage*), and phenological (LAI) and lepidochronological analyses (rhizome age), were carried out to assess the conservation status of *P. oceanica* meadows at Tremiti Islands MPA, understanding the ongoing changes and the effects of human activities. The latter embrace both anthropogenic impacts and positive effects due to conservation initiatives at a local scale (e.g., the establishment of mooring buoy fields), providing indications for appropriate management measures.

## 2. Materials and Methods

**Figure 1:**
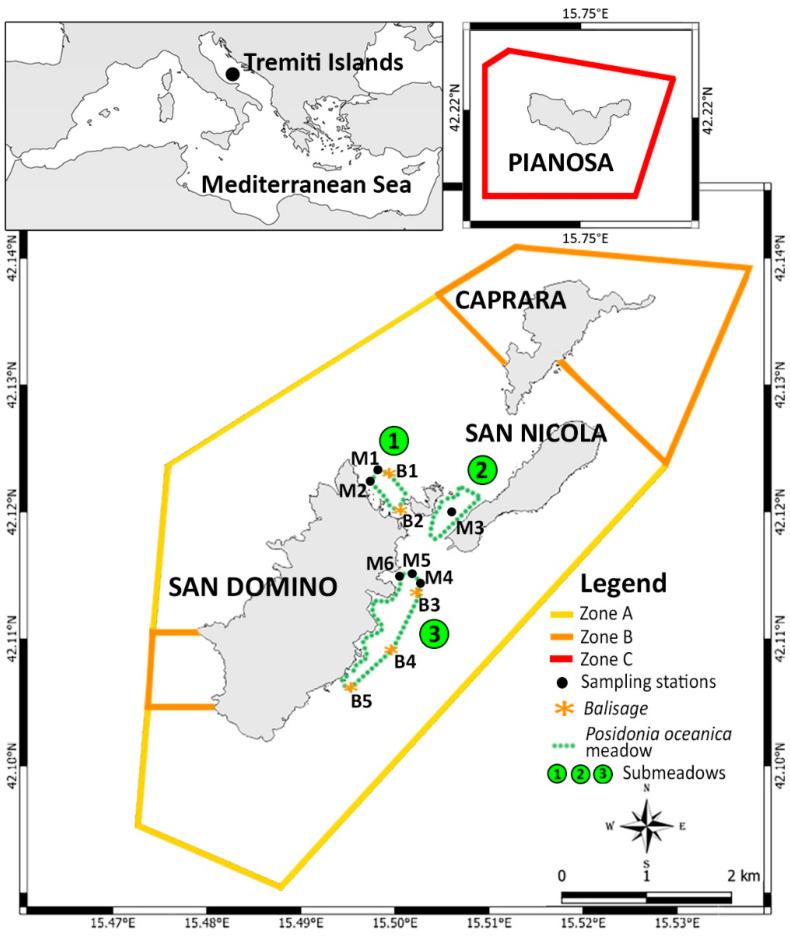
Map of the study area showing the sampling stations (M1–M6), the *balisage* sites (B1–B5), and the general distribution of *P. oceanica* meadow. Zone A (no take, no entry area, 12 nautical miles away from the three main islands), Zone B (highly protected area), and Zone C (partially protected area).

The study area is the Tremiti Islands MPA (42°7.38′ N–15°30.02′ E), located in the southern region of the Adriatic Sea, 12 nautical miles north of the Gargano Promontory (Apulia Region, Italy). The archipelago consists of three main islands called San Domino, San Nicola, and Caprara; a smaller island called Cretaccio; and a further island called Pianosa located 12 nautical miles northeast the three main islands. The seabed of the area is characterized by a conspicuous diversity of habitats and species, as well as a high aesthetic value [55,56,58]. The MPA is divided into three zones with different restrictions (Figure 1): Zone A (no take, no entry zone), where no human activity is allowed; Zone B (highly protected), where anchoring and recreational fishing are prohibited; and Zone C (partially protected), where only scientific research and professional fishing must be authorized, while all the other human activities can be practiced. *P. oceanica* is present only in Zone C [59], with a meadow fragmented in three main portions, here called submeadow 1 (located along the northeast coast of San Domino Island), submeadow 2 (located between San Nicola and Cretaccio Islands), and submeadow 3 (located along the east coast of San Domino Island) (Figure 1).

Six sampling stations (M1–M6) were monitored by scuba diving in June 2003, July 2015, and July 2020 (Figure 1; Table 1). Sampling stations were selected considering, when possible, the lower limit, the upper limit, and a middle zone at 15 m depth, according to national protocol by the Italian Institute for Environmental Protection and Research (ISPRA, available at www.isprambiente.gov.it; accessed on 6 March 2022). In detail, at submeadow 1 was considered the lower limit (M1, 19 m depth) and the upper limit (M2, 15 m depth), the latter coinciding with the depth of −15 m. Submeadow 2 was only studied in one central site (M3, 8 m depth) due to the lack of a clear upper/lower limit and the high rarefaction of the submeadow. Submeadow 3 was monitored at its lower limit (M4, 21 m depth), at the middle (M5, 15 m depth), and at the upper limit (M6, 8 m depth). The position and the depth of the sampling stations allocated in the proximity of the limits of the meadow changed slightly from 2003 to 2020 according to the progression or the regression of each limit. The percentage coverage of living *P. oceanica* in a 5 m radius area was estimated at each sampling station [4]; then absolute and relative rhizome density measures (rhizomes m^−2^) were carried out, with nine random replicates per station. The absolute rhizome density was estimated using a sampler frame of 40 × 40 cm; then it was expressed as rhizome m^−2^ [4]. The relative rhizome density was calculated by multiplying the absolute rhizome density for the percentage coverage. Both absolute and relative densities were expressed as mean ± standard deviation. Meadow areas were classified according to the meadow health scale proposed by Giraud [46], considering the following classes based on absolute density: class I (>700 bundles m^−2^, very dense meadow), class II (from 401 to 700 bundles m^−2^, dense meadow), class III (from 301 to 400 bundles m^−2^, sparse meadow), class IV (from 151 to 300 bundles m^−2^, very sparse meadow), and class V (from 50 to 150 bundles m^−2^, semi-meadow). Furthermore, meadow areas were classified according to Pergent et al. [47], which considers four classes based on absolute density and meadow depth: class ED (exceptional density), class ND (normal density, in balance meadow), class LD (low density, disturbed meadow), and class AD (abnormal density, very disturbed meadow). 

Orthotropic rhizomes (i.e., living perpendicularly to the bottom) were sampled by scuba diving at each sampling station in June 2003, July 2015, and July 2020 (Figure 1; Table 1). Eighteen rhizomes per sampling station were collected in the middle of the meadow (M3, M5), while only six rhizomes per station were collected in the proximity of lower (M1, M4) and upper limits (M2, M6) due to the higher fragility of these regions. The phenological analysis allowed the calculation of the leaf area index (LAI; m^2^m^−2^) by considering the mean leaf surface of each bundle (cm^2^ rhizome^−1^) and the relative density of the meadow (rhizome m^−2^) at each sampling station [4].

The thickness of the scales, such as the lignified basis of the old leaves remaining on the rhizome once the leaf falls in autumn [60,61,62], was measured. Its value along the rhizome typically oscillates between a minimum and a maximum, where the period between two minimums in the scale thickness represents a lepidochronological year, also known as a cycle [63]. Each bundle was aged by counting the number of cycles per rhizome.

Mapping of the meadow was carried out in July 2015 and 2020 using a side-scan sonar (Klein 3000), acquiring simultaneously with a double frequency of 100 and 500 kHz. Positional data were provided by a Hemisphere Crescent R-Series dGPS, and water sound velocity was obtained using a Seabird SBE 21 device. Data were processed using CARIS SIPS 8 software, then imported into a GIS environment using ArcView 10.2 for interpretation and cartography. Mapping was supported by ground-truthing based on images collected by scuba diving.

Five *balisage* systems (B1–B5; Figure 1 and Table 1) were put in place in July 2015 to monitor the progression or the regression of the lower limits of the meadow in the presence of a uniform limit (i.e., not patchy). Submeadow 2 was not monitored due to lack of clear limits. The *balisage* technique consisted of the positioning of fixed objects (called *balises*) into the substrate along the lower limit of the meadow to assess its variation over time. The progression towards deeper areas generally indicates the meadow’s healthy status, which tends to grow and increase its surface, while the regression of the lower limit highlights stress conditions [64]. Each system consisted of five metal pickets of 1.5 m in length, implanted for half of their length in the seabed at a known distance from the lower limit and spaced about 5 m from each other. Each picket was numbered and labeled, then monitored again in July 2020. The changes in the *P. oceanica* lower limit over 5 years were evaluated by comparing the distance between each picket and the meadow limit [4,57,64].

A synopsis of the parameters considered during the monitoring is reported in Table 2.

Statistical analyses were carried out using Past 4.03 software. For each monitored variable (i.e., cover percentage, absolute density, relative density, LAI, number of cycles per rhizome), a test for equal variances was run to verify the variance homogeneity of the replicates. The Shapiro–Wilk W test was used to determine the normality of the data distribution. The parametric test *t* was run to test the statistical significance of the observed differences when data distribution yielded a normal result (Shapiro–Wilk W test, *p* > 0.05). On the contrary, the Kruskal–Wallis test was used when data distribution was not normal (*p* < 0.05).

## 3. Results

### 3.1. Meadow Density and Coverage

**Figure 2:**
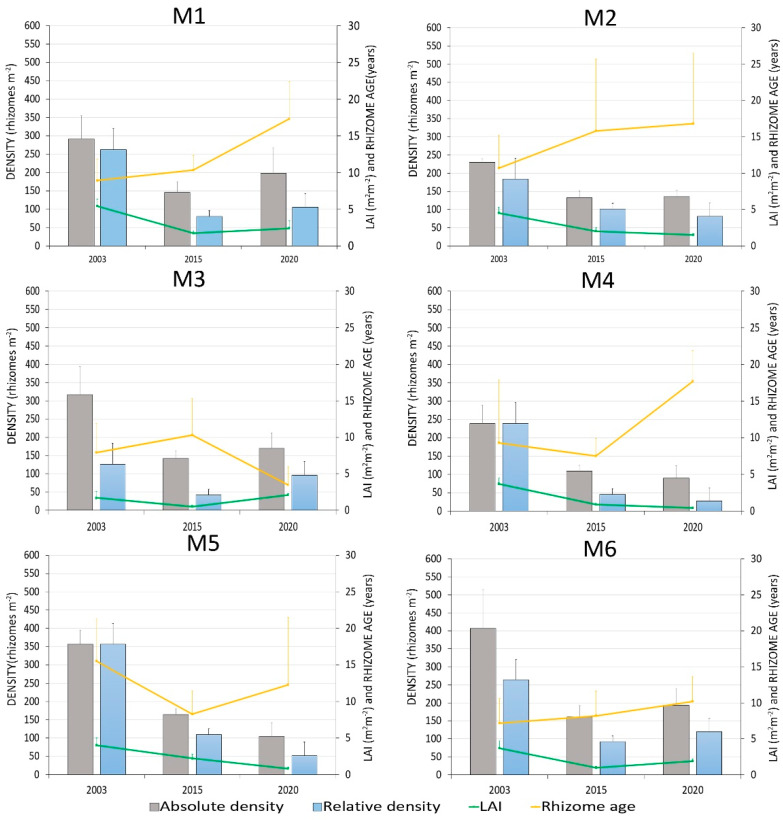
Variations in absolute and relative rhizome density (rhizomes m^−2^), leaf area index (LAI, m^2^ m^−2^), and rhizome age (lepidochronological years) of *Posidonia oceanica* at the Tremiti Archipelago in 2003, 2015, and 2020. Data represented as mean ± standard deviation. Information about each sampling station is reported in Table 1 and the text.

The absolute density of *P. oceanica* rhizomes decreased by about 40–60% between 2003 and 2015 in all the monitored sites (Figure 2 and Table 3 and Table S1). From 2015 to 2020, absolute density further decreased at the lower limit and the central portion of submeadow 3 (stations M4 and M5), while it had a stationary result or slightly improved in the other submeadows.

Relative density, such as rhizome density related to the percentage coverage, followed a similar trend with a significant decrease in all sites from 2003 to 2015 and a marked decrease at submeadow 3 (M4, M5) up to 2020. An increase in relative density was observed within 2015–2020, particularly in the presence of mooring buoys and reduced mechanical impacts (station M3), but also at the upper limit (station M6), and in areas of expansion of the meadow at the lower limit (station M1; see Section 3.3). Despite local improvements, the overall absolute and relative density values decreased significantly from 2003 to 2020 in all the monitored sites (Table 3).

**Figure 3:**
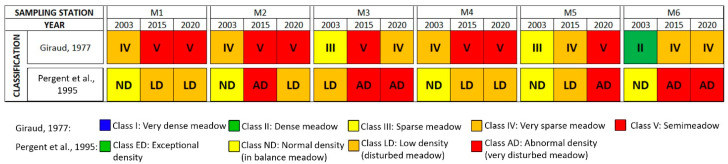
Classification of the meadow according to Giraud [46] and Pergent et al. [47].

The classifications proposed by Giraud [46] and by Pergent et al. [47] supported the general trend observed, showing a clear worsening of the status of the meadow from 2003 to 2015 and a slight improvement from 2015 to 2020 in certain areas (Figure 3).

### 3.2. Phenological and Lepidochronological Analysis

Between 2003 and 2015, also the amount of leaf area, indicated by LAI, strongly decreased throughout the meadow (Figure 2 and Table 3). This index continued to drop significantly up to 2020 at stations M4 and M5, where anthropogenic stressors were present (see Section 3.3). On the contrary, LAI improved in the presence of positive actions, such as the establishment of mooring buoy field mitigating mechanical impacts (station M3), at the upper limit (station M6), and, slightly, in areas of expansion of the meadow (station M1). The overall LAI significantly decreased throughout the meadow from 2003 to 2020, except at M3 (Figure 2 and Table 3).

Between 2003 and 2015, the mean number of cycles per rhizome (rhizome age) showed a significant decrease in the central part of submeadow 3 (M5), highlighted by the presence of young rhizomes. The other differences observed within the lepidochronological analysis were not significant (Figure 2 and Table 3). From 2015 to 2020, mean rhizome ages significantly increased at the lower limit of the meadow (M1 and M4), while at submeadow 2 (M3), the number of cycles per rhizome was significantly lower. Considering the overall monitored period, from 2003 to 2020, the mean number of cycles per rhizome showed a significant rise at the lower limit (M1 and M4) and a significant diminishing at submeadow 2 (M3). The differences in rhizome age concerning the upper limits (M2 and M6) and intermediate depth (M5) from 2003 to 2020 were not significant (Figure 2 and Table 3).

### 3.3. Expansion/Regression of P. oceanica and Anthropogenic Stressors

**Figure 4:**
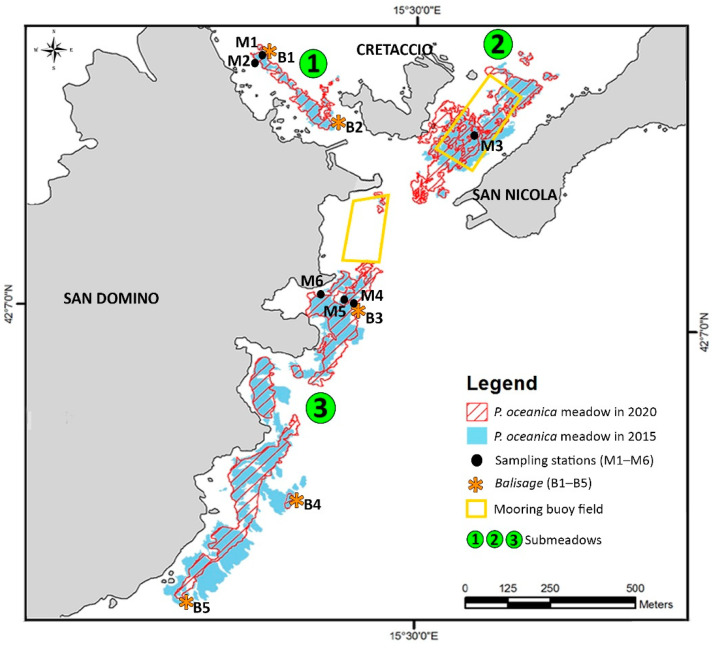
Map of the *Posidonia oceanica* meadow at the Tremiti Islands in 2015 and 2020. Sampling stations, *balisage* systems, and mooring buoy fields are also reported.

**Figure 5:**
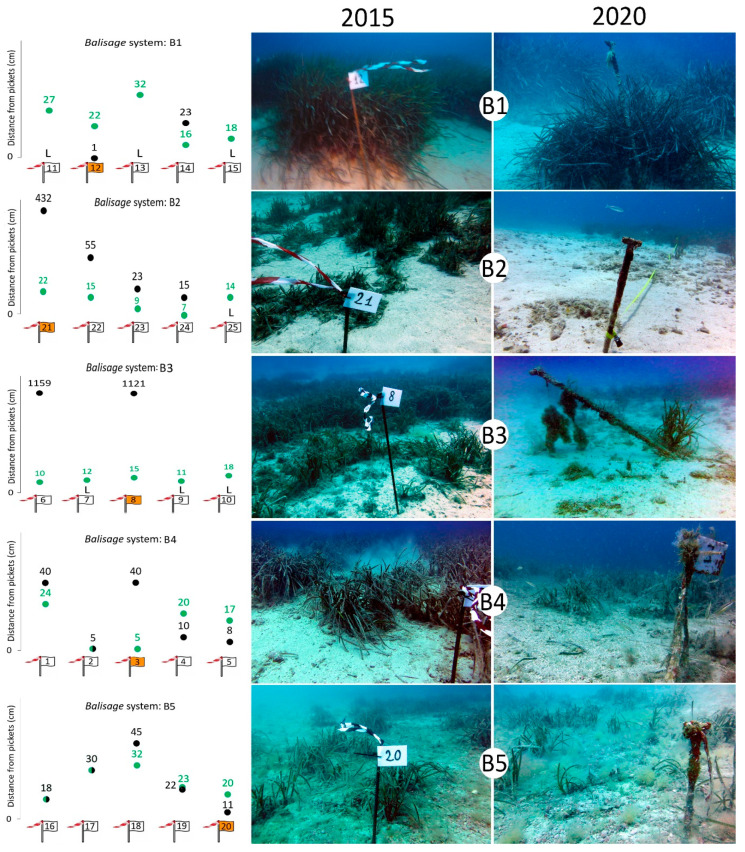
Graphic illustration of the distances between each picket and *Posidonia oceanica* in 2015 (green dots) and 2020 (black dots), with relative distances in cm. The orange flags represent the pickets shown on the right, as example of one of the five pickets at each *balisage* system. L: picket lost in 2020.

**Figure 6:**
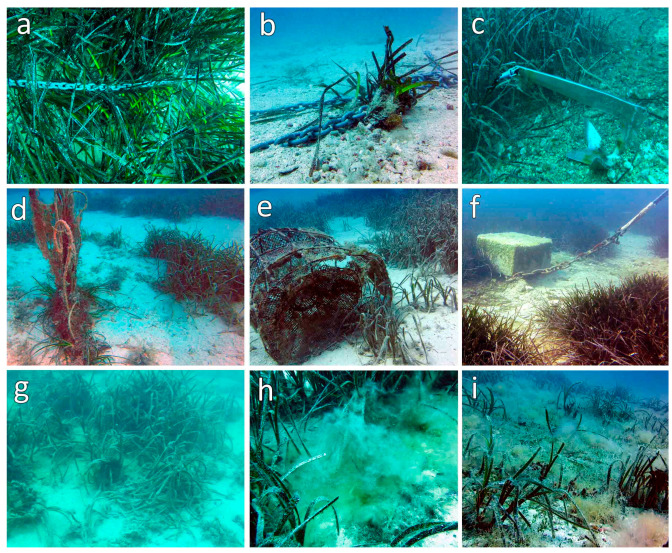
Main anthropogenic impacts affecting *Posidonia oceanica* at the Tremiti Islands. (**a**) Anchor chains on the meadow; (**b**) baring of *P. oceanica* bundles by anchor chain; (**c**) anchor on the meadow limit; (**d**) lost gillnet; (**e**) lost fishing trap; (**f**) abrasion of the chain on the seabed in a mooring system without a subsurface float between the anchor and the surface buoy; (**g**) burial of living bundles due to sedimentation; (**h**) mucilage on *P. oceanica*; (**i**) settlement of *Lophocladia lallemandii* on a degraded *P. oceanica* meadow.

The *P. oceanica* meadow of Tremiti Islands MPA covered a total surface of 156,347 m^2^ in 2015 and 134,154 m^2^ in 2020, with a 16.5% reduction of its area in 5 years (Table 4 and Figure 4). In detail, submeadow 1 showed a contraction on its southern side and an extension on its northern one, with a general surface increase of 5.6% from 2015 to 2020 (Table 4). This observation was also supported by the *balisage* system (Table 5 and Figure 5), which showed a marked regression from 8 to 410 cm on the southern side (*balisage* B2). On the northern side (*balisage* B1), most of the pickets were lost, and those still present were highly deformed. In this meadow area, several anchor scars (up to 2 scars m^−2^) and some lost anchors were observed (Figure 6). These impacts were less visible on the southern side where, on the other hand, a gillnet was found entangled in one of the pickets, attesting to the occurrence of artisanal fishing practices in the area.

Submeadow 2 showed a moderate surface increase of 6.2% in 5 years, particularly on its west and southwest sides. Anthropogenic mechanical stressors included the uneven presence of fish traps and pots, as well as visible abrasion by mooring chains in 2003 and 2015 (Figure 6). The latter were much less evident in 2020.

Submeadow 3 has undergone a fast surface reduction of 33.1% in 5 years, particularly at its lower limit (Table 4 and Figure 4). The only two pickets found at the northeast *balisage* system (B3), about 11 m away from the meadow limit (Table 5 and Figure 5), attested to a drastic contraction of this meadow area. Much farther to the south, the distance between the *balisage* systems (B4 and B5) and the closest living bundles of *P. oceanica* did not vary significantly, but the meadow was much rarefied and deteriorated in 2020 compared with 2015 (Figure 5). Only a few living bundles were present close to the *balisage* systems B4 and B5, which were several meters away from the marked limit of the meadow. All along this submeadow, *P. oceanica* was highly affected by sedimentation, mucilages, alien algal species such as the rhodophyte *Lophocladia lallemandii* (Montagne) F. Schmitz, and, at its northeast side, frequent anchoring activities carried out despite the presence of a mooring buoy field since 2017.

## 4. Discussion

The observed overall decline of *P. oceanica* meadow at the Tremiti Islands is strongly related to certain direct impacts including human activities on the coast, fishing, and anchoring practices. In fact, in the case of submeadow 3, a progressive sedimentation of the seabed, likely related to a change in the hydrodynamic regime near the harbor arm implemented and extended in the early 2000s, represented the main cause of the observed deterioration. Evidence of an increase in sedimentation rates due to the reduced hydrodynamism was highlighted within local studies, which showed a variation in the seabed granulometry [65,66]. In particular, the implant substrate of the meadow was mostly gravelly in 2003 [65], while a predominant fraction of very fine sand was observed in 2020 [66]. Besides the burial of the bundles, the increase in the sedimentation rate causes leaf shading, leaf abrasion, and lower photosynthetic efficiencies, together with the reduction of the exchanges of nutrients, CO_2,_ and O_2_, with the concomitant decrease in rhizome density and coverage [3,4]. This hypothesis is supported by the slightly better conditions observed at the upper limit, where the effects of the increased sedimentation are much less evident due to higher hydrodynamism. Moreover, anchoring practices and other anthropogenic impacts affected this submeadow especially on its northeast side, as attested by the absence of most pickets at the B3 *balisage* site and the deformed shape of the remaining ones. These actions significantly contributed to the contraction of *P. oceanica* at its lower limit, by causing mechanical impacts, such as rhizome baring and leaf damages, as well as enhancing sedimentation [14,23]. Concomitant biological phenomena related to global warming and organic pollution, such as algal blooms and massive mucilage production, act synergistically with direct human activities affecting *P. oceanica* meadows, as also observed in deeper areas [67]. The seabed left uncovered by *P. oceanica* can be suddenly colonized by invasive algae, such as the rhodophyte *Lophocladia lallemandii*, which compete for space [68]. Although present, artisanal fishing activities using gillnets, traps, and pots were much less important in terms of impacts compared with anchoring practices.

Due to its higher exposure to north-coming currents, submeadow 1 was much less impacted by sedimentation, while massive anchoring practices were the main anthropic cause of the worsening of this meadow area observed especially from 2003 to 2015 and mainly concerning the reduction of rhizome density and meadow coverage. Anchoring affected this submeadow especially on the northern side, as attested by several scars in the meadow, some lost anchors, and the lack of the majority of the pickets at the B1 *balisage* site, most likely due to anchoring activities. In fact, this area is highly appreciated by tourists for bathing and snorkeling activities, being frequented by a great number of recreational boats every year.

Submeadow 2 also showed an alarming worsening of its health status between 2003 and 2015. Indeed, this submeadow was historically used by many local boats because of its sheltered position between two islands, being highly affected by anchoring practices and showing strong signs of deterioration over time. However, a promising recovery rate was observed since 2015, with several improvements in terms of area, coverage, rhizome density, and LAI. The presence of young rhizomes in 2020 highlighted an ongoing stolonization process and the concomitant expansion of the submeadow, confirming the improvement of its general conservation status. This positive response of the submeadow has been triggered by effective mitigation actions, aimed at the removal of the main local impact represented by anchoring. Indeed, a mooring buoy field with a subsurface float system was installed in 2017, avoiding anchoring practices in correspondence with this meadow area and preventing, by means of the float system, chain abrasion on the seabed. Mitigation actions proved to be much less successful when the main impacts were not completely removed, as in the case of submeadow 3, where the installation of a mooring buoy field did not support the recovery of the meadow, still affected by high sedimentation rates hindering any improvement of its health state. 

The Tremiti Archipelago is characterized by a high diversity of habitats and species due to its position in the Adriatic Sea, benefiting from north-coming water masses, which bring nutrients from the northern part of the basin [69,70]. These peculiar oceanographic conditions support different habitats of great conservation value, including coral forests, rhodolith beds, and, at shallower depths, *P. oceanica* meadows [57,58,71]. Unfortunately, these habitats are currently suffering due to global warming, and heat-related mass mortality events have been recently reported at the Tremiti Islands, where extensive populations of the red gorgonian *Paramuricea clavata* (Risso, 1826) are largely affected by a combination of stressors, including heat waves, lowering of the thermocline, mucilaginous bloom, and massive macroalgal overgrowth [66,69,72]. This evidence related to global warming might also support the overall depletion of *P. oceanica* observed in our study [18,19,59,60,61], and is likely to be enhanced or mitigated by local conditions. In fact, local factors seem to have a great influence on the status of *P. oceanica* and its overall health state [21,36,37,73]. For these reasons, it is essential to regulate human activities in coastal zones and to avoid local destructive actions, especially in the presence of vulnerable habitats, such as seagrass meadows, in order to mitigate the effects of global anthropogenic impacts.

## 5. Conclusions

An overall decline of *P. oceanica* meadow was observed at the Tremiti Islands in the last 20 years. Locally, where proper mitigation actions have been adopted (mooring buoy field establishment) and significant adverse impacts removed, this habitat showed encouraging signs of improvement. Although the effects of global changes are difficult to contrast, the improvement of local conditions through appropriate management actions proved to be a successful strategy for the conservation of sensitive habitats, such as *P. oceanica* meadows, enhancing their resilience. The mitigation of anchoring practices and the regulation of coastal development, together with monitoring activities and appropriate dissemination campaigns to inform tourists and sea workers about how to mitigate the impacts on *P. oceanica* meadows, can play a crucial role in the conservation of this coastal habitat.

## Figures and Tables

**Table 1:** ID, coordinates, and depth of sampling stations and *balisage* systems for each submeadow.

ID	Type	Submeadow	Coordinates	Depth (m)
M1	Lower limit	1	42°07′25.73″ N–15°29′39.67″ E	19
M2	Upper limit	1	42°07′24.85″ N–15°29′38.92″ E	15
M3	Middle zone	2	42°07′18.12″ N–15°30′9.36″ E	8
M4	Lower limit	3	42°07′0.26″ N–15°29′52.05″ E	21
M5	Middle zone	3	42°07′0.43″ N–15°29′50.62″ E	15
M6	Upper limit	3	42°07′1.17″ N–15°29′46.74″ E	8
B1	*Balisage*	1	42°07′26.04″ N–15°29′40.37″ E	19
B2	*Balisage*	1	42°07′18.63” N–15°29′48.59″ E	10
B3	*Balisage*	3	42°07′00.13″ N–15°29′53.22″ E	21
B4	*Balisage*	3	42°06′38.92″ N–15°29′35.71″ E	22
B5	*Balisage*	3	42°06′28.75″ N–15°29′26.07″ E	25

**Table 2:** Synopsis of the parameters considered during each monitoring and analysis performed to assess each parameter. Dots were used to indicate that the parameters were calculated during monitoring.

Parameters	Analysis and Purpose	2003	2015	2020
Absolute density (rhizomes m^−2^)	Number of living rhizomes present in a 40 × 40 cm sampler frame to quantify the number of rhizomes per surface unit	•	•	•
Relative density (rhizomes m^−2^)	Absolute rhizome density in relation to the percentage coverage as an indicator of the actual density of the whole meadow	•	•	•
Leaf area index (m^2^m^−2^)	Mean leaf surface of each bundle in relation to the relative density to assess the potential photosynthetic surface of the meadow	•	•	•
Rhizome age (years)	Number of lepidochronological cycles per rhizome, identified by two thickness scale minimums, to assess the age of the rhizomes, with particular attention to the occurrence of young rhizomes as an indication of an active meadow renovation	•	•	•
Habitat mapping	Side-scan sonar survey to assess the distribution and the extension of the meadow		•	•
*Balisage systems*	Measurement of distances between pickets and meadow limits over time to assess any progression or regression of the meadow		•	•

**Table 3:** Differences in *p*-values (*t*-test) between different years of monitoring within each sampling station.

ID	Year	Absolute Rhizome Density (Rhizomes m^−2^)	Relative Rhizome Density (Rhizomes m^−2^)	Leaf Area Index (m^2^m^−2^)	Rhizome Age (Years)
M1	2003 vs. 2015	6.25 × 10^−5^ ***	4.52 × 10^−5^ ***	6.38 × 10^−7^ ***	0.171
	2015 vs. 2020	0.053	0.149	0.156	0.013 *
	2003 vs. 2020	0.038 *	6.1 × 10^−3^ *	1.0 × 10^−3^ *	2.6 × 10^−3^ *
M2	2003 vs. 2015	2.31 × 10^−7^ ***	8.40 × 10^−8^ ***	1.32 × 10^−5^ ***	0.172
	2015 vs. 2020	0.773	4.0 × 10^−3^ *	0.071	0.863
	2003 vs. 2020	1.79 × 10^−7^ ***	3.87 × 10^−10^ ***	8.76 × 10^−7^ ***	0.102
M3	2003 vs. 2015	2.64 × 10^−5^ ***	3.13 × 10^−6^ ***	6.0 × 10^−3^ *	0.296
	2015 vs. 2020	0.087	5.67 × 10^−6^ ***	3.24 × 10^−8^ ***	0.013 *
	2003 vs. 2020	5.11 × 10^−4^ ***	0.060	0.278	0.029 *
M4	2003 vs. 2015	8.09 × 10^−6^ ***	5.01 × 10^−8^ ***	1.06 × 10^−6^ ***	0.424
	2015 vs. 2020	0.148	3.60 × 10^−4^ ***	1.41 × 10^−5^ ***	4.58 × 10^−4^ ***
	2003 vs. 2020	2.10 × 10^−5^ ***	2.32 × 10^−8^ ***	1.68 × 10^−7^ ***	4.2 × 10^−3^ *
M5	2003 vs. 2015	1.15 × 10^−8^ ***	2.79 × 10^−10^ ***	2.60 × 10^−6^ ***	1.3 × 10^−3^ *
	2015 vs. 2020	3.65 × 10^−4^ *	4.41 × 10^−7^ ***	5.89 × 10^−10^ ***	0.622
	2003 vs. 2020	4.98 × 10^−8^ ***	1.14 × 10^−10^ ***	6.97 × 10^−13^ ***	0.212
M6	2003 vs. 2015	2.87 × 10^−5^ ***	1.23 × 10^−5^ ***	3.481 × 10^−5^ ***	0.352
	2015 vs. 2020	0.096	0.030 *	3.95 × 10^−4^ ***	0.373
	2003 vs. 2020	2.11 × 10^−4^ ***	1.26 × 10^−4^ ***	2.0 × 10^−3^ *	0.172

* *p* < 0.05; *** *p* < 0.001.

**Table 4:** Submeadow and entire meadow surfaces in 2015 and 2020, with the indication of increase or decrease (%) estimated in 2020.

	Area (m^2^)		Area (ha)		Increase/Decrease (%)
Submeadow	2015	2020	2015	2020	
1	11,010	11,666	1.101	1.167	+5.6
2	42,291	45,083	4.229	4.508	+6.2
3	103,046	77,405	10.305	7.740	−33.1
Total	156,347	134,154	15.635	13.415	−16.5

**Table 5:** Distance (cm) between each of the five pickets per *balisage* system (B1–B5) and the closest *Posidonia oceanica* rhizomes at the lower limit of the meadow in 2015 and 2020. L: picket lost.

*Balisage*	Picket ID	2015 Distance (cm)	2020 Distance (cm)	Variation (cm)
B1	11	27	L	
	12	22	1	+21
	13	32	L	
	14	16	23	−7
	15	18	L	
B2	21	22	432	−10
	22	15	55	−40
	23	9	23	−14
	24	7	15	−8
	25	14	L	
B3	6	10	1159	−1149
	7	12	L	
	8	15	1121	−1106
	9	11	L	
	10	18	L	
B4	1	24	40	−16
	2	5	5	0
	3	5	40	−35
	4	20	10	+10
	5	17	8	+9
B5	16	18	18	0
	17	30	30	0
	18	32	45	−13
	19	23	22	+1
	20	20	11	+9

## Data Availability

The dataset generated during and/or analyzed for the current study is available from the corresponding author upon request.

## Supplementary Materials

The following are available online at https://www.mdpi.com/article/10.3390/biology11060923/s1, Table S1: Density, phenological, and lepidochronological results for each sampling station in 2003, 2015, and 2020.
