# Supplementary material for: The Status of Posidonia oceanica at Tremiti Islands Marine Protected Area (Adriatic Sea)
Source: Biology (Basel). 2022 Jun 16;11(6):923. doi: 10.3390/biology11060923 (PMC9220029; doi:10.3390/biology11060923)
Supplement: Supplementary file 1 [file biology-11-00923-s001.zip › biology-1650839-supplementary.pdf]

Article

The Status of *Posidonia oceanica* at Tremiti Islands Marine Protected Area (Adriatic Sea)

Andrea Tursi [1,2,*], Francesco Mastrototaro [1,2], Federica Montesanto [3], Francesco De Giosa [4], Anna Lisco [1], Antonella Bottalico [1] and Giovanni Chimienti [1,2]

1 Department of Biology, University of Bari Aldo Moro, 70125 Bari, Italy; francesco.mastrototaro@uniba.it (F.M.); annalisco87@gmail.com (A.L.); antonella.bottalico@uniba.it (A.B.); giovanni.chimienti@uniba.it (G.C.)
2 CoNISMa (Consorzio Nazionale Interuniversitario per le Scienze del Mare), 00196 Rome, Italy
3 University of Nebraska-Lincoln, 1400 R Street, Lincoln, NE 68588, USA; federica.montesanto@uniba.it
4 Environmental Surveys S.r.l. (ENSU), Via De Gasperi, 74123 Taranto, Italy; francescodegiosa@ensu.it
* Correspondence: andrea.tursi@uniba.it

Table S1. Density, phenological, and lepidochronological results for each sampling station in 2003, 2015, and 2020.

**Station ID**	**M1**			**M2**			**M3**			**M4**			**M5**			**M6**		
**Year**	2003	2015	2020	2003	2015	2020	2003	2015	2020	2003	2015	2020	2003	2015	2020	2003	2015	2020
**Depth (m)**	19	19	19	17	15	15	10	8	8	23	22	19	15	15	15	11	8	9
**Absolute rhizome density (rhizomes m^{-2})**	291.3 ± 63.5	145.8 ± 28.6	197.9 ± 69.1	230.0 ± 10.3	132.6 ± 19.2	135.2 ± 18.1	316.3 ± 76.9	141.7 ± 21.1	169.9 ± 41.4	238.8 ± 49.3	108.7 ± 16.4	89.6 ± 33.9	356.3 ± 38.0	164.9 ± 15.1	104.2 ± 37.6	406.3 ± 108.1	160.8 ± 31.7	193.8 ± 46.0
**Relative rhizome density (rhizomes m^{-2})**	262.1 ± 57.2	80.2 ± 15.7	105.6 ± 36.9	184 ± 8.2	101.7 ± 14.7	81.1 ± 10.9	126.5 ± 30.8	42.5 ± 6.3	96.3 ± 23.4	238.8 ± 49.3	45.3 ± 6.8	26.9 ± 10.2	356.3 ± 38.0	110.0 ± 10.0	52.1 ± 18.8	264.1 ± 70.2	92.7 ± 18.3	119.5 ± 28.4
**Leaf area index (m^2m^{-2})**	5.4 ± 1.0	1.7 ± 0.3	2.4 ± 1.0	4.5 ± 0.8	2.0 ± 0.5	1.5 ± 0.2	1.7 ± 0.9	0.5 ± 0.2	2.1 ± 0.2	3.7 ± 0.8	0.9 ± 0.1	0.4 ± 0.1	4.0 ± 1.0	2.2 ± 0.6	0.8 ± 0.2	3.7 ± 1.0	1.0 ± 0.2	1.9 ± 0.3
**Rhizome age**	8.9 ± 2.9	10.3 ± 2.0	17.3 ± 5.1	10.7 ± 4.5	15.8 ± 9.9	16.8 ± 9.7	7.9 ± 4.0	10.3 ± 5.0	3.5 ± 2.5	9.3 ± 8.6	7.5 ± 2.4	17.7 ± 4.2	15.5 ± 5.8	8.3 ± 3.2	12.3 ± 9.2	7.2 ± 3.4	8.2 ± 3.5	10.2 ± 3.5
